# Three-dimensional facial swelling evaluation of pre-operative single-dose of prednisone in third molar surgery: a split-mouth randomized controlled trial

**DOI:** 10.1186/s12903-023-03334-y

**Published:** 2023-08-31

**Authors:** Alessandro Antonelli, Selene Barone, Francesco Bennardo, Amerigo Giudice

**Affiliations:** 1 Department of Health Sciences, School of Dentistry, Magna Graecia University of Catanzaro, Viale Europa, 88100 Catanzaro, Italy; 2 Department of Health Sciences, Oral Surgery Residency Training Program Director, Dean of the School of Dentistry, Magna Graecia University of Catanzaro, Catanzaro, Italy

**Keywords:** Third molar surgery, Wisdom teeth, Three-dimensional facial swelling, Corticosteroids, Oral surgery, Prednisone, Pain, Inflammatory sequelae, Swelling, Trismus

## Abstract

**Background:**

Facial swelling, pain, and trismus are the most common postoperative sequelae after mandibular third molar (M3M) surgery. Corticosteroids are the most used drugs to reduce the severity of inflammatory symptoms after M3M surgery. This study aimed to evaluate the effect of a single pre-operative dose of prednisone on pain, trismus, and swelling after M3M surgery.

**Methods:**

This study was designed as a split-mouth randomized, controlled, triple-blind trial with two treatment groups, prednisone (PG) and control (CG). All the parameters were assessed before the extraction (T0), two days (T1), and seven days after surgery (T2). Three-dimensional evaluation of facial swelling was performed with Bellus 3D Face App. A visual analogue scale (VAS) was used to assess pain. The maximum incisal distance was recorded with a calibrated rule to evaluate the trismus. The Shapiro–Wilk test was used to evaluate the normal distribution of each variable. To compare the two study groups, the analysis of variance was performed using a two-tailed Student t-test for normal distributions. The level of significance was set at a = 0.05. Statistical analysis was conducted using the software STATA (STATA 11, StataCorp, College Station, TX).

**Results:**

Thirty-two patients were recruited with a mean age of 23.6 ± 3.7 years, with a male-to-female ratio of 1:3. A total of 64 M3Ms (32 right and 32 left) were randomly assigned to PG or CG.

Surgery time recorded a mean value of 15.6 ± 3.7 min, without statistically significant difference between the groups. At T1, PG showed a significantly lower facial swelling compared to CG (PG: 3.3 ± 2.1 mm; CG: 4.2 ± 1.7 mm; *p* = 0.02). Similar results were recorded comparing the groups one week after surgery (PG: 1.2 ± 1.2; CG: 2.1 ± 1.3; *p* = 0.0005). All patients reported a decrease in facial swelling from T1 to T2 without differences between the two groups.

At T1, the maximum buccal opening was significantly reduced than T0, and no difference between PG (35.6 ± 8.2 mm) and CG (33.7 ± 7.3 mm) (*p* > 0.05) was shown. Similar results were reported one week after surgery (PG: 33.2 ± 14.4 mm; CG: 33.7 ± 13.1 mm; *p* > 0.05). PG showed significantly lower pain values compared to CG, both at T1 (PG: 3.1 ± 1.5; CG: 4.6 ± 1.8; *p* = 0.0006) and T2 (PG: 1.0 ± 0.8; CG: 1.9 ± 1.4; *p* = 0.0063).

**Conclusion:**

Our results showed that pre-operative low-dose prednisone administration could reduce postoperative sequelae by improving patient comfort after M3M surgery and reducing facial swelling two days and one week after surgical procedures.

**Trial registration:**

www.clinicaltrials.gov — NCT05830747 retrospectively recorded—Date of registration: 26/04/2023.

## Introduction

Third-molar removal represents one of the most frequent procedures in oral surgery [1]. Although the complication rate is relatively low and transient, this surgical procedure is related to significant post-operative morbidities [2]. Surgical trauma is mainly due to a higher prevalence of bone impaction and cortical bone in the mandible compared to the maxilla and variable teeth orientation with different surgical risks [3–5]. Compared to traditional dental extractions, mandibular third molar (M3M) surgery frequently involves post-operative inflammatory nature symptoms such as swelling, pain, and trismus [6, 7]. These symptoms should be managed and reduced as much as possible to improve the patient’s quality of life. For this purpose, many therapeutic approaches have been suggested, such as non-steroid anti-inflammatory (NSAIDs) drugs or corticosteroids administration, proteolytic agents, cryotherapy, autologous platelet concentrate application, low-level laser therapy and surgical drains [8–13]. Among the pharmacological therapies, corticosteroid drugs are the most commonly preferred pharmaceutical agents for reducing the severity of the inflammatory symptoms after surgical removal of M3M [14]. The action of corticosteroids decreases the inflammatory phases by inhibiting the phospholipase A2 pathway. It limits the synthesis of prostaglandins, leukotrienes, and thromboxane A2-related substances. In addition, corticosteroids limit the release of lysozyme and the vasodilating action of bradykinin, reducing edema [15]. According to their duration of action, corticosteroids are classified into short-acting, intermediate-acting, and long-acting (Table 1) [16]. Although it has been reported that long-acting corticosteroids have a better effect in controlling post-operative complaints, there is evidence that long-term administration and massive dosing can induce alterations in the hypothalamic–pituitary–adrenal (HPA) axis [17, 18]. Other studies have shown that short-term therapy with short- or intermediate-acting drugs can be just as effective [8, 19]. In addition to the type of drug, the time and mode of administration play an essential role in managing post-operative inflammation [20, 21]. Although many researchers suggested one treatment choice over another, there is no consensus on the ideal therapy to reduce post-surgical sequelae after M3M removal.

**Table 1:**
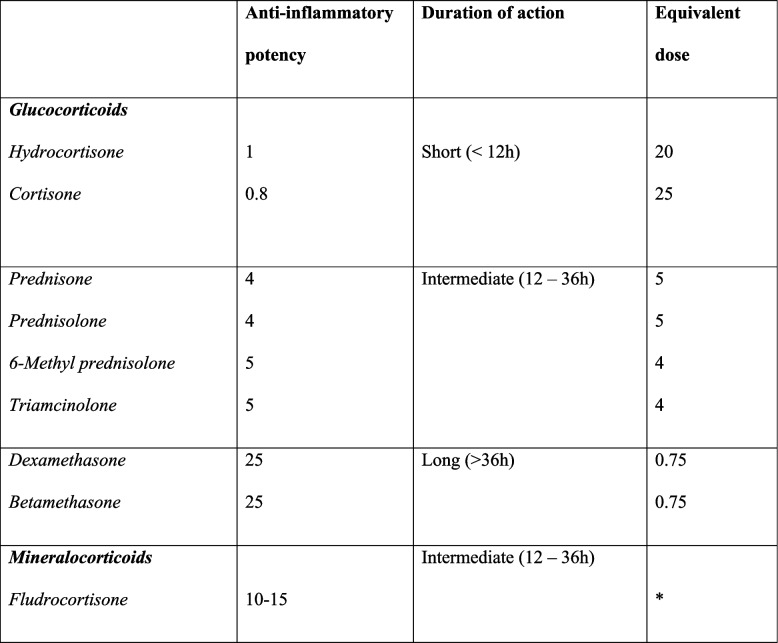
The anti-inflammatory potency and duration of action of corticosteroids

*Glucocorticoids with a mineralocorticoid effect roughly equivalent to 0.1 mg fludrocortisone are: prednisone or prednisolone 50 mg, or hydrocortisone 20 mg

This study aimed to compare the effect of a single preoperative dose of prednisone versus placebo in terms of facial swelling, trismus, and pain after surgical removal of M3M in a split-mouth randomized controlled clinical trial. Facial swelling was evaluated using an innovative three-dimensional digital technique.

## Materials and methods

The present article is reported according to the CONSORT statement and its extension for within-person randomized trials [22].

### Study design

All methods were carried out in accordance with relevant Declaration of Helsinki. The regional Ethical Review Board of Central Calabria “Regione Calabria—Comitato etico sez. area centro” (reference for Magna Graecia University of Catanzaro) approved the study (n. 465/2020). The study was designed as a randomized split-mouth study (www.clinicaltrials.gov — NCT05830747 retrospectively recorded—Date of registration: 26/04/2023).

### Study sample

Participants were recruited in the Academic Hospital of Magna Graecia University of Catanzaro, Italy. According to the inclusion criteria, patients aged 18 to 32 years who required both M3M extractions were recruited. The inclusion criteria were as follows: good health status; indication to surgical extraction of both M3M; complete root formation (STAGE H, Donald B. Shumaker) [23]; surgical risk level classified as “Conventional” or “Moderate” according to Daugela et al. classification [24]. The exclusion criteria were as follows: person under the age of 18 or over 32; allergy or contraindications to administration of corticosteroids; acute infection in any of the teeth to be extracted; patients with chronic liver disease, diabetes, immune system dysfunction, or haematological disease; pregnancy or breastfeeding; history of treatment with antiresorptive drugs; chronic kidney disease; history of systemic corticosteroid therapy in the past 4 weeks. Informed consent was obtained from all patients enrolled after being adequately informed on the risks and the potential benefits of the treatment. All patients signed a consent for the publication of data or photos for scientific purposes.

### Sample calculation and randomization

The sample size was calculated given an effect size of 0.5. With a power of 85% and a type I error of 0.05, 29 patients would have been needed. The randomization was processed using a computer-generated random shortlist. Each treatment (test and control) was assigned to the specific site (left or right M3M) by choosing between identical, opaque envelopes containing different possible combinations. One operator, not involved in surgeries, knew the assignments and administered drugs to the patients accordingly.

### Procedure

**Fig. 1:**
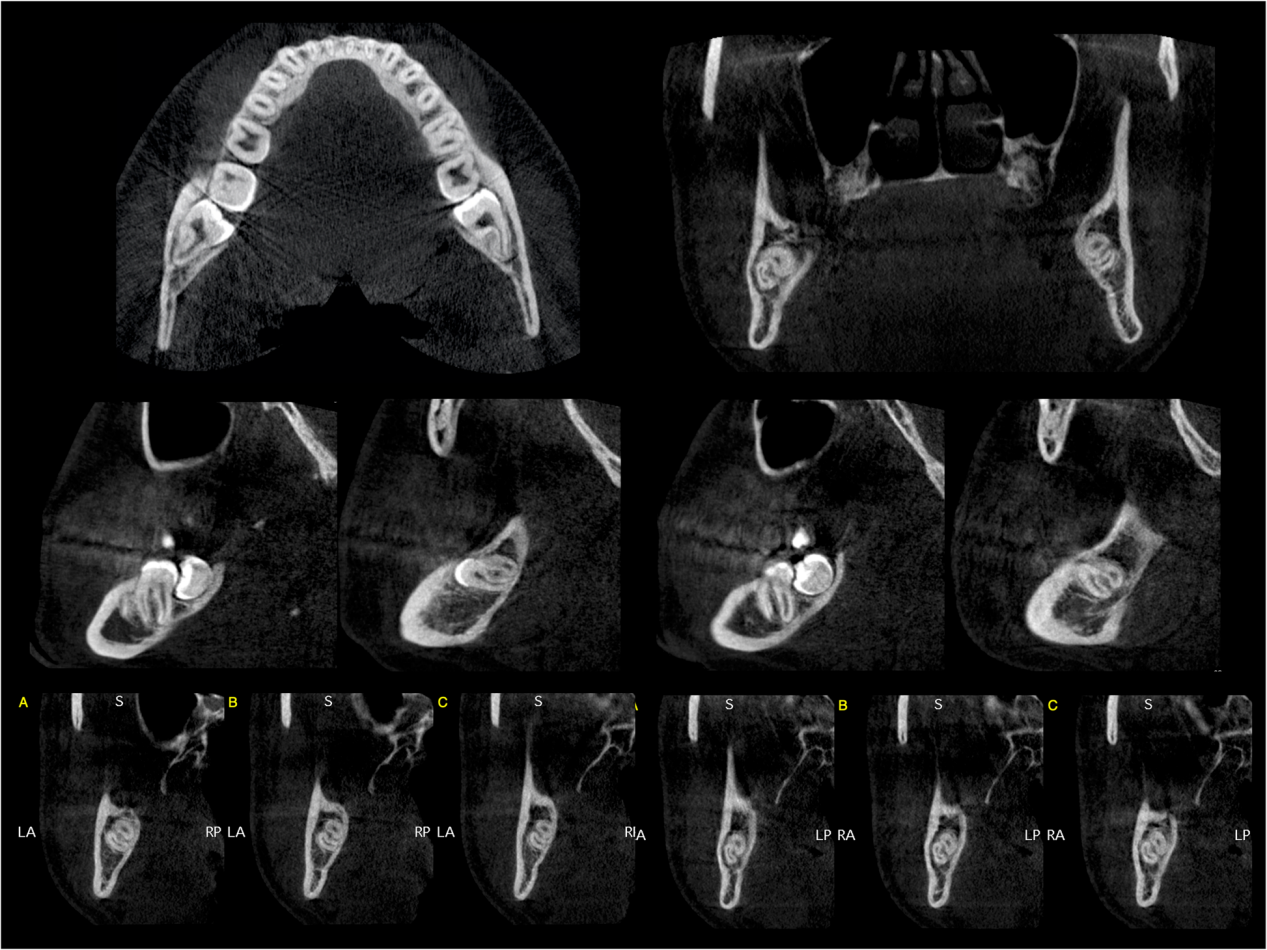
A pre-surgical evaluation with CBCT scan was performed to assess the presumptive surgical risk for each M3M

After enrollment, all patients underwent professional oral hygiene. A pre-surgical cone beam computed tomography (CBCT; X-Mind® Trium, Acteon®, Mérignac, FR) was performed to assess M3M surgical intervention risk according to Daugela et al. (Fig. 1) [24].

All patients enrolled in the study were treated by a single certified oral and maxillofacial surgeon (AG), unaware of randomization. During test side surgery, patients received, in a randomized manner, a pre-operative single dose of prednisone 25 mg (Deltacortene 25 mg, Bruno Farmaceutici, Roma, IT; PG – prednisone group) or placebo (CG – control group) per os, 1 h before third molar extraction.

Surgeries were performed four weeks apart.

A single dose of antibiotic prophylaxis was administered 1 h before surgery: 2 g of amoxicillin or 600 mg of clindamycin in case of allergy. Immediately before surgery, the patient rinsed with a 0.20% chlorhexidine gluconate solution (Curasept, Curaden HealthCare, Italy) for one minute. Preoperative antibiotic prophylaxis and the use of local antiseptics for all patients aimed to reduce the bacterial load as much as possible near surgery.

**Fig. 2:**
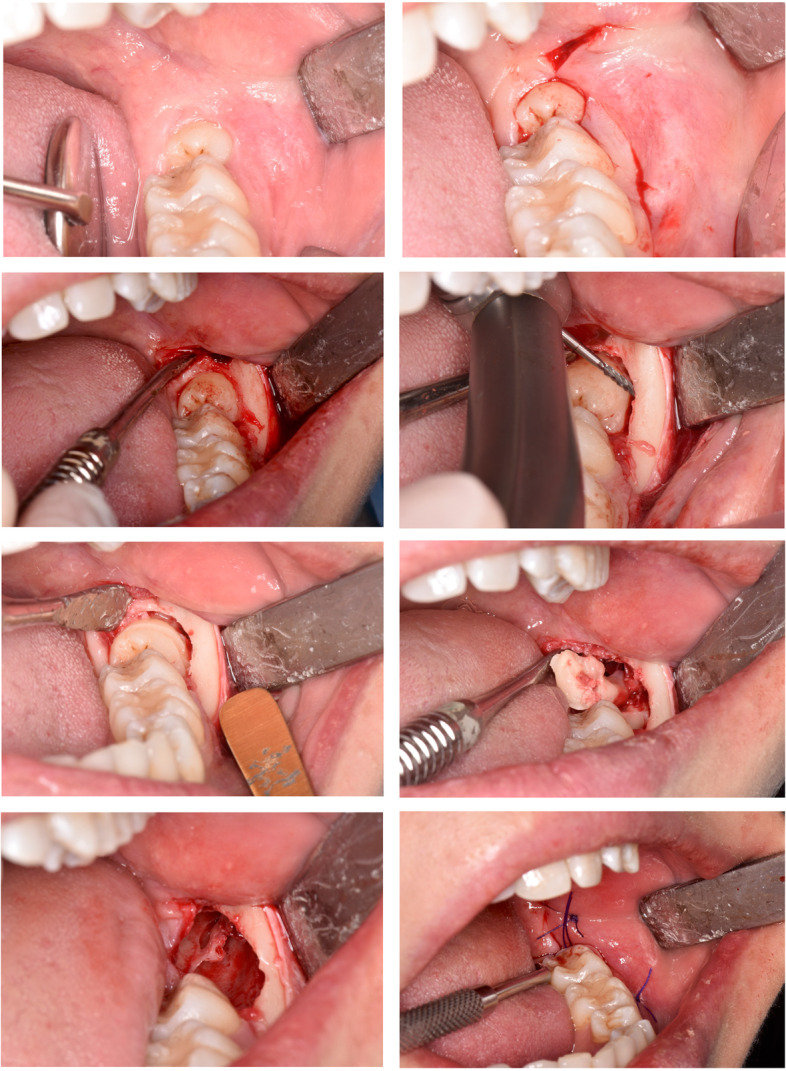
Surgical sequence of M3M (3.8) removal. All surgical procedures were performed by the same experienced oral surgeon following a strict schedule

**Fig. 3:**
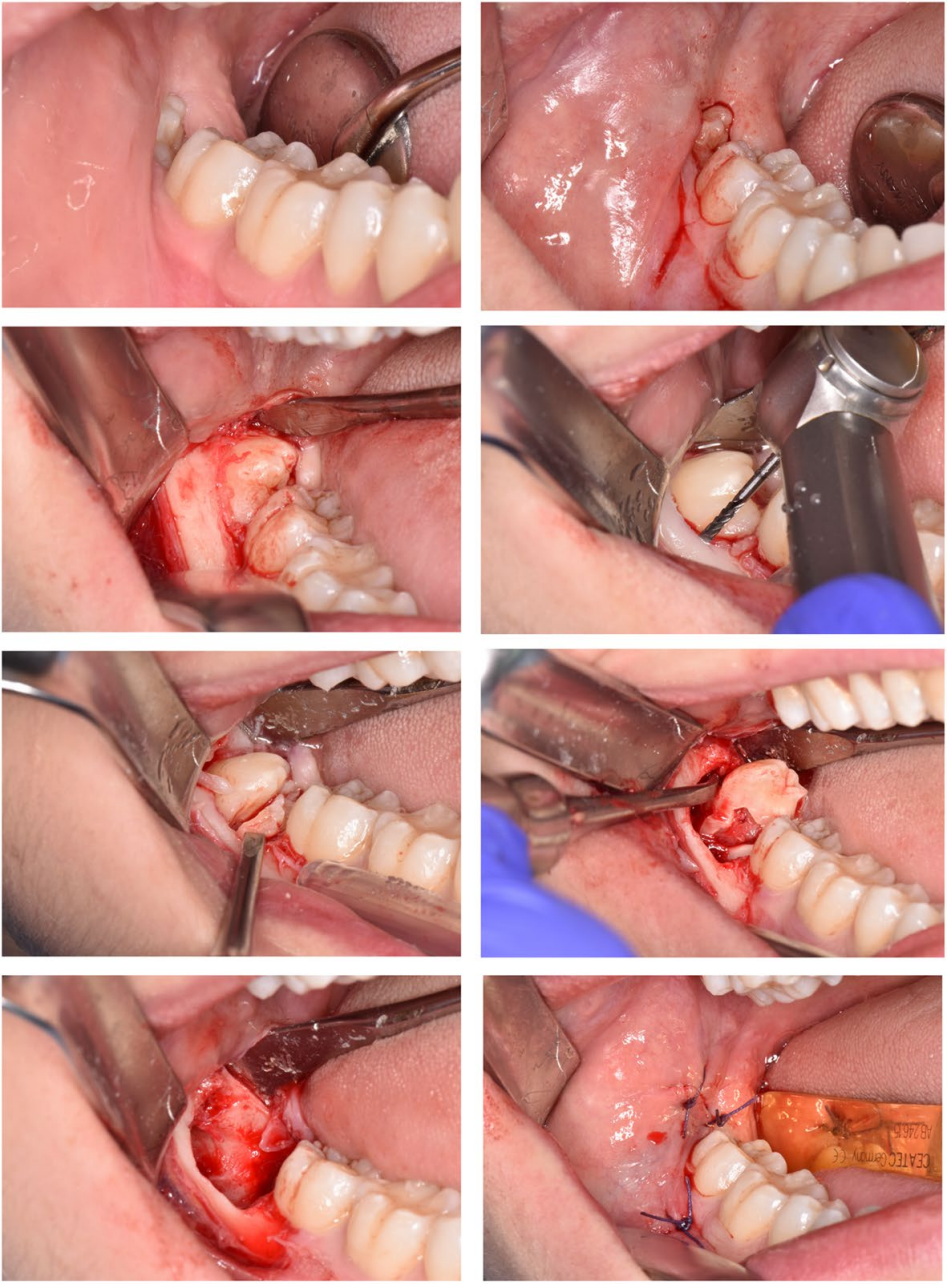
Surgical sequence of M3M (4.8) removal. All surgical procedures were performed by the same experienced oral surgeon following a strict schedule

Local anesthesia with mepivacaine 2%/epinephrine 1:100.000 (Optocain, Molteni Dental, Italy) was performed to obtain inferior alveolar, lingual, and buccal nerves block. Ten minutes after anesthesia, the surgery started with the elevation of a mucoperiosteal modified-triangular flap. Osteotomy was performed with a straight low-speed handpiece and round bur (Komet Dental, Lemgo, Germany) to expose M3M, and the tooth was sectioned using a highspeed handpiece and fissure bur (Komet Dental, Lemgo, Germany) under continuous irrigation with sterile saline solution. When required, the root separation was performed, and the M3M was subsequently removed. The surgical site was revised and irrigated with physiological saline solution, and then the flap was repositioned and sutured using a 3–0 resorbable suture (Vicryl, Ethicon, United States). Figures 2 and 3 report one case as an example.

An independent operator recorded the time (in minutes) for each surgical M3M removal.

Time of surgery was considered from the start of incision to the end of suturing.

**Fig. 4:**
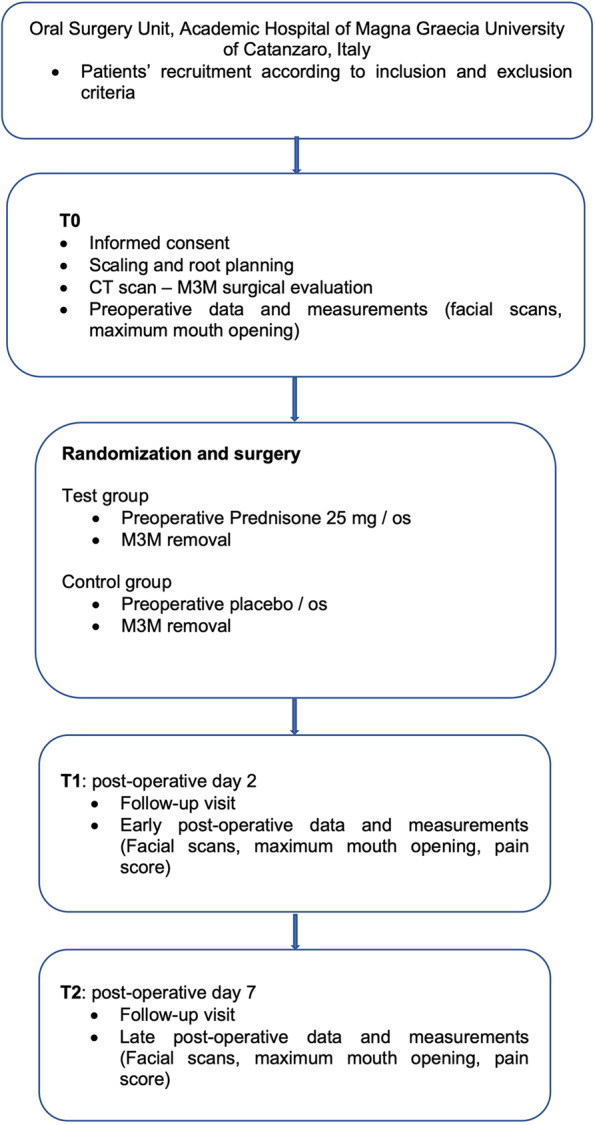
Flow chart of study procedures

Each patient received the same post-operative instructions: paracetamol 1 g three times a day for two days starting immediately after surgery; rinsing with 0.2% chlorhexidine mouthwash for at least 1 min twice a day for one week, starting the day after surgery. The follow-up visits were scheduled two (T1) and seven (T2) days after surgery. Any other drug intake or adverse event after surgery was recorded. The summarized study protocol is presented in Fig. 4.

### Three-dimensional assessment of facial swelling

**Fig. 5:**
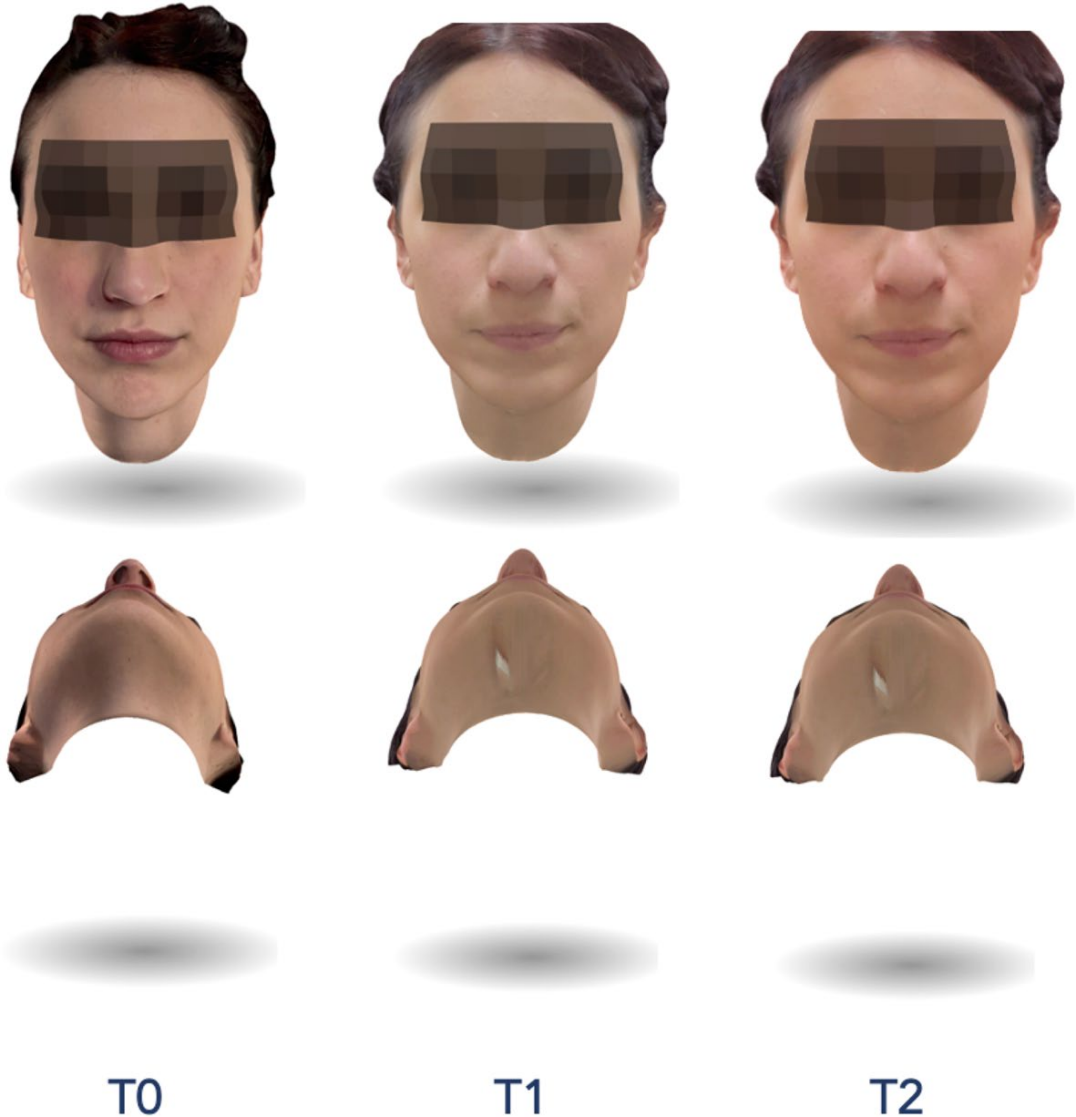
Assessment of facial swelling using a 3D face scan 3 times for M3M (3.8): immediately before the intervention (T0), 2 days (T1) and 7 days after surgery (T2)

**Fig. 6:**
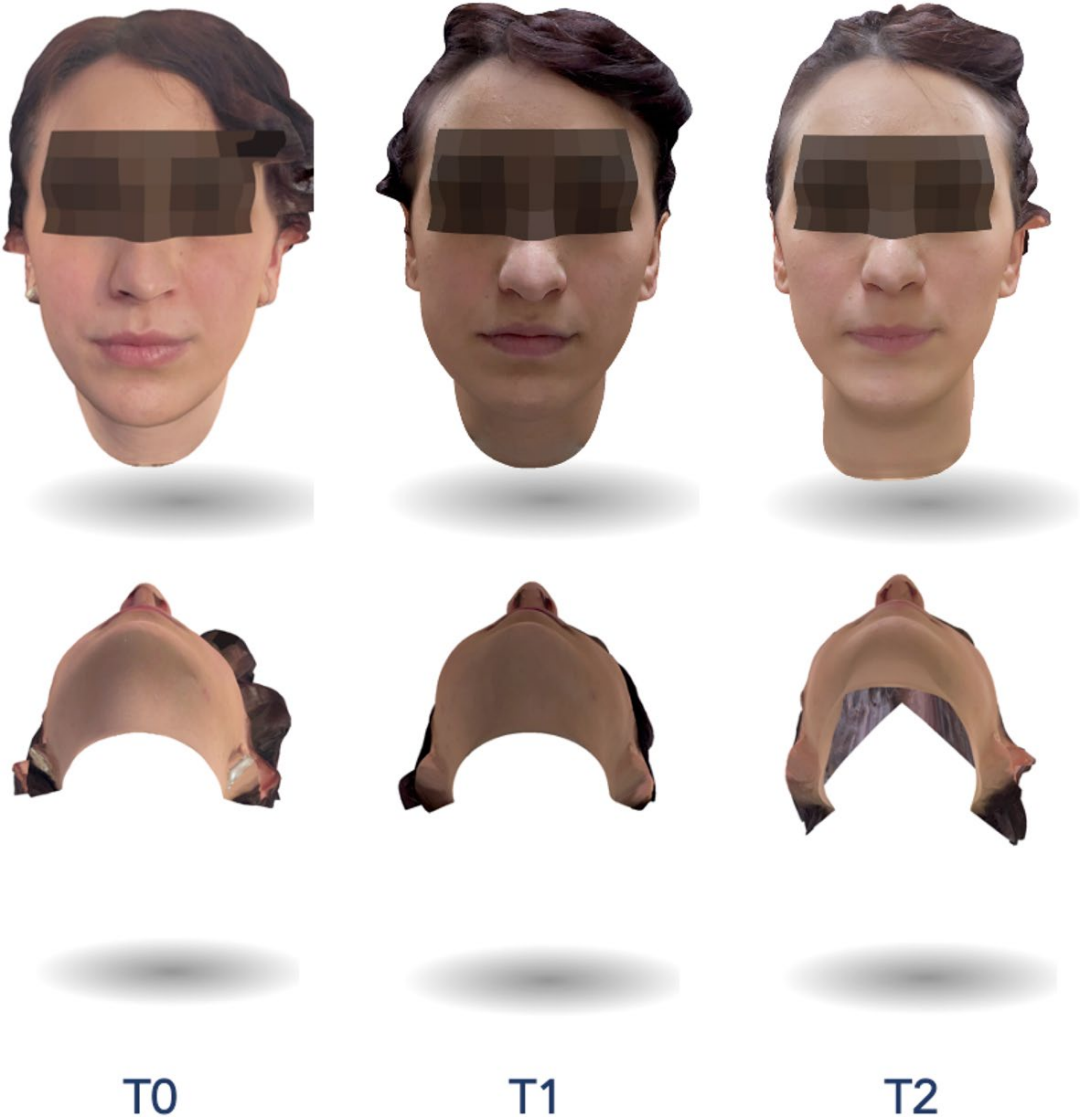
Assessment of facial swelling using a 3D face scan 3 times for M3M (4.8): immediately before the intervention (T0), 2 days (T1) and 7 days after surgery (T2)

To assess the facial swelling, patients performed a 3D face scan 3 times for each M3M surgery: immediately before the intervention (T0), 2 (T1) and 7 (T2) days after surgery (Figs. 5 and 6). All facial scans were recorded in the same room and under the same lighting conditions. Bellus3D Face Camera Pro System© (model number FCP01, Bellus 3D Face App, Bellus3D Inc.) available for iOS (iPhone 12, Apple Inc. CA, USA) was used to capture facial scans. This application provided a simple face guided scan. At the end of the procedure, an STL file of the face scan was generated. The scan type we set up was a 24 MB HD file with 250,000 triangles and a 4 K color texture map.

The STL files were imported into the software 3D Slicer (3D Slicer, version 4.11.20210226, Brigham and Women's Hospital, Harvard University, NIH) and a superimposition of the pair of scans was performed (T0-T1 and T1-T2 and T0-T2) using “Model to Model Distance” specific tool. The “Shape Population Viewer” plug-in allowed to achieve a colormap for the qualitative evaluation. Using the “Pick'n Paint” plug-in, the Region of Interest (ROI) was selected in each facial scan, considering the tragus of the ear as the upper limit, soft tissue gonion as the lower, cheilion and parasymphyseal region as the medial limit, in accordance with Lau et al. [25]. The amount of the millimetric differences between the two surfaces were recorded using the “Mesh Statistics” tool.

### Assessment of maximum mouth opening

Maximum mouth opening was recorded at each follow-up visit using a calibrated rule. The maximum inter-incisal distance between the upper and lower central incisor was calculated.

### Assessment of pain

Pain intensity was assessed using a Visual Analog Scale (VAS) with score from “0 – No pain” to “10 – Worst pain”. Post-operative pain was recorded after surgery (T0) and at scheduled follow-up visits (T1 and T2).

### Statistical analysis

Data were collected in spreadsheets (Excel, Microsoft Office, Redmond, US). Treatment site (test or control) was the primary predictor variable. Descriptive statistics recorded mean and standard deviation for continuous quantitative variables, absolute and relative frequencies for categorical data. Shapiro–Wilk test was used to evaluate the normal distribution of each variable. To compare the two study groups, the analysis of variance was performed using a two-tailed Student t-test for normal distributions. Alternatively, the non-parametric Wilcoxon test was performed. Linear regression analysis allowed to correlate the outcomes variable with the surgical time. The level of significance was set at a = 0.05. Statistical analysis was conducted using the software STATA (STATA 11, StataCorp, College Station, TX).

## Results

### Study sample

**Fig. 7:**
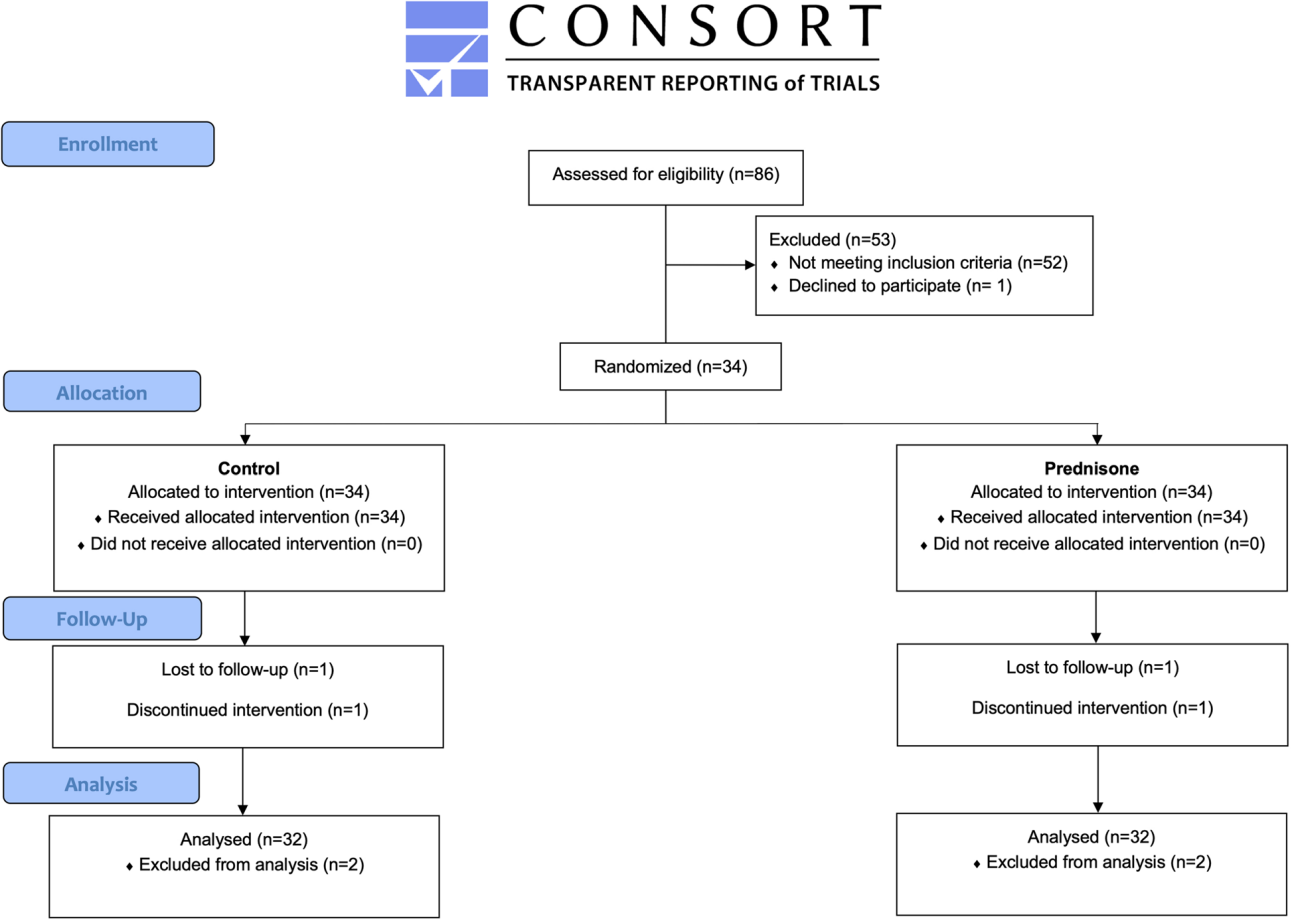
CONSORT diagram illustrate that 86 patient were eligible, out of these 34 patients were randomly allocated in two groups. Two patients were not available for data analysis

During the clinical trial period, 86 patients were recruited, but only 34 met the inclusion criteria. One patient withdrew, and another patient was excluded because of missed follow-up visits. A total of 32 patients were included in the study sample (Fig. 7). The mean age of patients was 23.6 ± 3.7 (range 20–28 years). Twenty-four were female and eight males, with a male-to-female ratio of 1:3. Sixty-four M3M surgeries were analyzed (32 right M3M and 32 left M3M) and randomly assigned to PG or CG. Demographic data are reported in Table 2. Surgery time recorded a mean value of 15.6 ± 3.7 min, without statistically significant difference between the groups (PG: 15.7 ± 3.9 min; CG: 15.6 ± 3.5 min; *p* > 0.05). No postoperative infections or haemorrhagic complications or nerve damage were observed during the follow-up period.

**Table 2:**
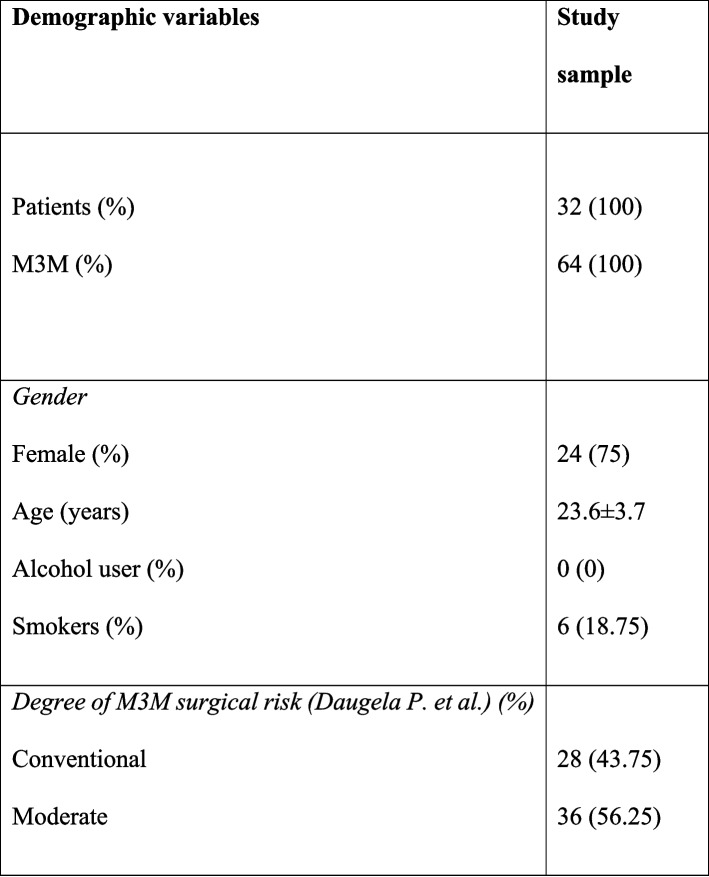
Study population baseline characteristics

### Qualitative analysis

**Fig. 8:**
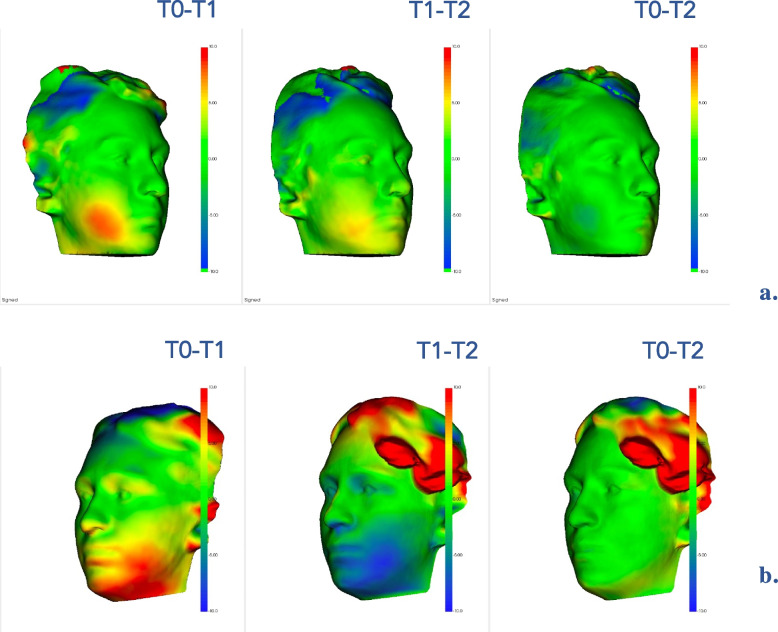
Qualitative analysis of the facial swelling scans at different time-points: T0-T1; T1-T2; T0-T2 in the TG (**a**). T0-T1; T1-T2; T0-T2 in the CG (**b**). Red colour represented an increase of facial swelling in the ROI, while blue colour indicated a decrease of it. In the CG, the region of interest (ROI) reported a more extensive red area including the oral commissures zone at T0-T1. In T1-T2 superimposition a greater decrease of facial swelling was recorded in PG compared to CG; a marked blue region is appreciated in CG due to higher initial face edema at T1. In the comparison of T0-T2 facial scans, no clinical difference can be appreciated in PG while a little edema may persist in the colormap of the CG (yellow zone)

Qualitative analysis is reported in Fig. 8, showing semitransparent overlays of facial scans at different timings (T0-T1, T1-T2, and T0-T2). The most significant discrepancy was recorded comparing facial swelling at T1 with the baseline (T0).

### Quantitative analysis—inflammatory outcomes evaluation

#### Facial swelling

All patients showed an increase in facial swelling after surgery compared to T0 (T0-T1: 3.8 ± 2 mm; T0-T2: 1.7 ± 1.3 mm). According to the primary predictor variable, at T1, PG showed a significantly lower facial swelling compared to CG (PG: 3.3 ± 2.1 mm; CG: 4.2 ± 1.7 mm; *p* = 0.02). Similar results were recorded comparing the groups one week after surgery (PG: 1.2 ± 1.2; CG: 2.1 ± 1.3; *p* = 0.0005). All patients reported a decrease in facial swelling from T1 to T2; however, no statistically significant differences have been found between the groups (PG: 2.1 ± 1.5; CG: 2.0 ± 1.3; *p* = 0.7).

Linear regression showed a significant correlation between facial swelling and surgery time. In particular, longer surgical time was related to higher facial swelling at both T1 and T2 (*p* < 0.001).

#### Maximum mouth opening

**Fig. 9:**
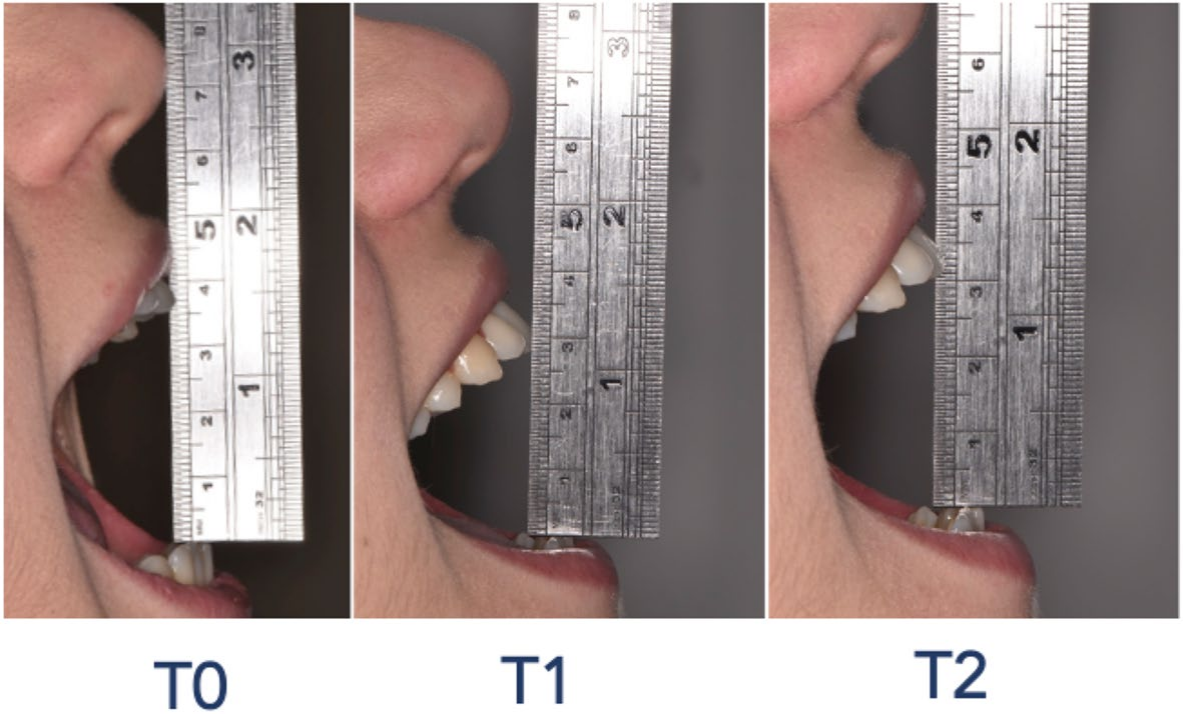
Assessment of maximum mouth opening at T0 – T1 – T2 timeline using a calibrated rule. The maximum inter-incisal distance between the upper and lower central incisor was calculated

Before surgery, all patients showed good values of buccal opening (44.9 ± 4.6 mm; PG: 44.9 ± 4.5 mm; CG: 44.8 ± 4.8 mm). At T1, the maximum buccal opening was significantly reduced with no difference between PG (35.6 ± 8.2 mm) and CG (33.7 ± 7.3 mm) (*p* > 0.05). The inflammatory effect persisted one week after surgery in both groups (PG: 33.2 ± 14.4 mm; CG: 33.7 ± 13.1 mm; *p* > 0.05) (Fig. 9).

#### Pain

All patients showed low values of local pain at T0 (0.83 ± 1.2; PG: 0.75 ± 1.2; CG: 0.9 ± 1.1). After surgery, the pain trend increased at T1 (3.9 ± 1.8) and then reduced at T2 (1.4 ± 1.2). According to the primary predictor variable, PG showed significantly lower pain values compared to CG, both at T1 (PG: 3.1 ± 1.5; CG: 4.6 ± 1.8; *p* = 0.0006) and T2 (PG: 1.0 ± 0.8; CG: 1.9 ± 1.4; *p* = 0.0063).

## Discussion

Tissue damage arising from surgical trauma in M3M removal often results in common postoperative inflammatory sequelae, such as swelling, trismus, and pain, which negatively affect the patient's quality of life, especially immediately after surgery [2]. A literature review highlights that several different approaches have been adopted to reduce postoperative inflammatory symptoms [8–13]; among these, the use of corticosteroid drugs has been widely and deeply analyzed, and their potential benefits on the inflammatory process have been assessed [14, 16]. However, which route of administration and dosage is best for the patient remains an open question [16].

In this split-mouth randomized clinical trial, we analyzed the effects of the pre-operative use of prednisone (25 mg/os) on inflammation sequelae caused by M3M surgical removal in a cohort of 32 patients. Data were collected at two different time points, specifically at 2 and 7 days, after surgery. This study's primary outcome was to compare the efficacy of prednisone/os vs placebo in reducing facial swelling using three-dimensional qualitative and quantitative analysis. The secondary outcome was to assess the effects of prednisone/os administration on trismus and pain.

Although both groups experienced facial swelling after the removal of M3M, patients treated with prednisone (PG) showed significantly less facial swelling and pain at both T1 and T2 compared to the placebo sample (CG). However, the T1-T2 evaluation showed a greater reduction in facial swelling compared to the control group. No significant associations with trismus were observed. It is important to note that these results were unbiased both for the invasiveness and the length of the surgical procedure, as no differences between the two study groups existed in terms of surgical risk stratification (Daugela et al.) and surgical time of M3M removal. In line with our data, Tiigimae-Saar et al. conducted a prospective study showing that the administration of prednisolone can be effective in reducing edema, especially in the first four days following M3M removal surgery [19]. Similarly, Buyukkurt et al. reported that a single dose of 25 mg prednisolone could be effective compared to a placebo in reducing facial edema at 2 and 7 days follow-up [26]. The combination of low doses of prednisolone (10 mg) with NSAIDs can also reduce edema following third-molar surgery compared to taking NSAIDs or placebo alone [27]. In contrast with these papers, Kang et al. pointed out that pre-emptive administration of 10 and 20 mg prednisolone per/os was not sufficient to improve postoperative inflammatory symptoms [28]. However, the lack of significance found in this study could be due to the limited sample size and subjective data collection. Postoperative symptoms were assessed using a questionnaire in which patients reported changes in symptoms for six days after the surgical procedure [28].

In our study, we selected prednisone for the pre-surgical therapeutic approach. Prednisolone and prednisone belong to glucocorticoids and share the same mechanism of action [16]. Both drugs are rapidly absorbed after oral administration, reaching peak plasma concentration after 1 to 3 h. However, the plasma half-life of prednisone is slightly longer (3.4 to 3.8 h) than that of prednisolone (2.1 to 3.5 h) [29].

In line with preliminary studies by Kim and Yuasa, our correlations between surgery duration and facial swelling showed that a longer surgical time was significantly associated with larger edema both at T1 and T2 in the groups, with no difference between PG and CG [30, 31]. This phenomenon can be attributed to increased vascular permeability due to tissue damage and greater surgical stress [32, 33].

Pain assessment showed an increase in symptoms at T1, followed by a subsequent decrease at T2 for both PG and CG. Comparing the two patient groups, PG reported less pain than CG at both time points. According to our results, oral prednisone administration one hour before surgery was correlated with lower pain compared to placebo, especially seven days after surgery, thus improving the patient's quality of life. As shown by Acham et al., a single oral administration of methylprednisone 1 h before surgery is significantly effective in monitoring postoperative pain during the first week of follow-up [8]. These data are supported by a recent systematic review with meta-analysis where the administration of corticosteroid therapy was reported to be more effective in reducing postoperative pain compared to placebo [34].

Evaluation of maximum mouth opening revealed a significant decrease at T1, regardless of patient groups. Despite symptom improvement for PG and CG, postoperative inflammation continued to have a negative effect on mouth opening at follow-up T2 with no significant differences between the two groups. These findings are in agreement with Buyukkurt et al., who previously reported that although prednisolone administration results in a clear improvement in pain, it is not associated with better effects on post-surgical trismus than placebo [26]. Although these data are partially controversial compared to other studies in which trismus is often closely related to pain, it is important to emphasize that in our study, the participants continued to have small degrees of pain and alterations in mouth opening at the end of the follow-up.

This evidence is reported by several authors who point out an improvement in postoperative sequelae by taking corticosteroids with incomplete disappearance of symptoms such as pain and trismus beyond one week of follow-up [25, 35]. Indeed, as reported by Pedersen et al., there is only sometimes a strong correlation between improvement in facial swelling, pain, and trismus after M3M surgery [36].

The efficacy of corticosteroid-based therapy in reducing post-surgical discomfort in oral surgery has been widely investigated in the literature [14].

A literature review on M3M surgery from 2000 to 2020 clearly highlighted a high interest in the scientific research around the administration of corticosteroids and around i) the most appropriate type of drug, ii) the best administration route, and iii) the most effective dosage for the management of post-surgical inflammatory symptoms and for the patient's quality of life improvement [37]. In this context, dexamethasone (DM) and methylprednisone (MP) corticosteroid drugs appeared to be the most studied ones due to their glucocorticoid properties and minimal mineralocorticoid effects [8].

As reported in several studies, the short duration of action and moderate dosage of MP are sufficient to manage inflammatory symptoms while avoiding possible systemic complications. However, many authors prefer the use of DM due to its greater anti-inflammatory potency, its prolonged drug effect (> 36 h), or the repeated administration of steroids in the following days [38, 39]. This prevents a possible rebound of swelling on the second and third postoperative days. In this regard, data arising from our study showed that the use of prednisone did not cause any rebound of swelling. Indeed, in the case of surgical removal of M3M with conventional/moderate difficulty, short-term therapy with a single pre-operative administration of prednisone appeared to be effective, avoiding multiple and prolonged administrations as well as possible alterations of the HPA axis [18].

So far, several administration routes for corticosteroids have been proposed, including intravenous, intramuscular, submucosal, and oral. The intravenous route of administration shows the advantage of leading to a rapid increase of drug concentration within plasma; intramuscular and submucosal administration provide encouraging data on the treatment efficacy. However, the former is characterized by slow onset and increased risk of HPA axis disruption, while the latter may cause local adverse events such as tissue necrosis or abscess at the injection site [40, 41]. The enteral absorption of prednisone and dexamethasone has effectively reduced postoperative inflammatory symptoms [14, 16, 42]. From this perspective, the oral route of administration may be preferable and well tolerated by patients compared to the intravenous route.

As previously reported, the best dosage depends on the type of corticosteroid used. Despite this general concept, most of the available studies showed that a high dosage effectively manages inflammatory sequelae due to oral surgery procedures.

While Mojsa et al. showed that the submucosal administration of 4 mg Dexamethasone is necessary to manage the M3M comorbidities, Erdil et al. suggested a double dosage equal to 8 mg [17, 43]. Buyukkurt et al. indicated that the administration of 25 mg of prednisolone was well-suited to control swelling, pain, and trismus after M3M surgery procedures, while in a prospective, randomized, placebo-controlled, double-blind study with a split-mouth design, Acham et al. showed that the optimal dosage methylprednisolone for the management of M3M surgical removal-related symptoms was 40 or 80 mg [8, 26].

Moreover, in a review by Ngeow WC et al., it is reported that to achieve the same effects of 10 mg prednisolone, it is necessary to prescribe 8 mg methylprednisolone or 50 mg cortisone, or 40 mg hydrocortisone (cortisol), or 1.5 mg betamethasone or dexamethasone [16]. This suggests that the choice of drug to be used is patient-dependent and very often falls to the clinical experience of the practitioner.

The results of our study suggest that pre-emptive short-term therapy of low doses (25 mg/os) of prednisone is effective compared to placebo in the management of postoperative symptoms caused by M3M removal. This concept is in accordance with a recent systematic review suggesting that pre-operative administration of corticosteroids is clinically preferable, but without identifying the optimal dosage and route of administration [44, 45].

Overall, our study shows strengths but, at the same time, weaknesses that need to be kept in mind in order to analyze the results correctly. Starting with the strengths, to our knowledge, this is the first study evaluating the efficacy of prednisone administration prior to M3M removal through the correlation of surgical difficulty assessed by CT scan-based grading and through the analysis of outcomes with 3D facial imaging technology.

Moreover, the use of very strict inclusion criteria for the enrollment of patients, as well as the triple-blind clinical study design, are certainly other major strengths of our study.

The splith-mouth design used in our study (i.e. bilateral removal of M3M with similar position and degree of surgical risk in the same patient) is quite similar to other splith-mouth randomized clinical trials [8, 12, 13, 20, 46, 47]. The main purpose of this model is to reduce all components related to differences between the subjects examined using a cross-over study.

Furthermore, the assessment of facial edema using 3D scans is more predictable and accurate than other empirical procedures [46, 48]. Indeed, over the years, several methods have been used to assess facial edema in oral and maxillofacial surgery, but these are often inaccurate and operator-dependent [49–52]. Although 3D stereophotogrammetry is a new approach in the evaluation of facial edema after M3M surgery, the results proposed by several studies seem to be encouraging in defining the region affected by swelling [25]. Still, face scanners can be expensive and not always available. From this point of view, the use of smartphone apps that exploit 3D stereophotogrammetry can overcome the cost problem while providing equally valid and reproducible results [53]. As assessed in many studies evaluating different three-dimensional facial scans, the use of Bellus3D FaceApp allowed us to obtain results with a high degree of accuracy at a low cost and easily reproducible [54, 55].

In particular, as highlighted by Dzelzkaleja et al., who analyzed the performance of several apps, Bellus3D FaceApp is effective in keeping face scans compliant, with simple and fast image acquisition, avoiding the deformation of the scanned model [56].

On the other hand, our study has some limitations, mainly represented by the small sample examined, including only M3Ms with simple or moderate surgical risk. A further limitation might be the lack of an objective method of pain assessment.

## Conclusion

The results showed in this split-mouth randomized clinical trial assessed that pre-operative administration of prednisone is adequate to reduce postoperative sequelae by improving patient comfort after M3M surgery with conventional/moderate surgical difficulty.

Patients undergoing third molar surgery under local anesthesia may benefit from a single administration of low-dose prednisone. Furthermore, this administration route is simple, convenient, and comfortable for both patient and surgeon.

The digital analysis performed in our study allowed a more objective evaluation of the facial swelling, reducing costs and possible bias.

However, the authors believe that further multicenter randomized controlled trials are necessary to validate this treatment's efficacy and to extend the results at all types of M3M.

## Data Availability

The data that support the findings of this study are available on request from the corresponding author.

## Abbreviations

M3M: Mandibular third molar
NSAIDs: Non-steroid anti-inflammatory drugs
PG: Prednisone group
CG: Control group
HPA: Hypothalamic–pituitary–adrenal axis
DM: Dexamethasone
MP: Methylprednisone
